# Glutathione Impacts Both *Batrachochytrium dendrobatidis* Virulence and Amphibian Cellular Defense in a Chytridiomycosis Model

**DOI:** 10.1002/mbo3.70271

**Published:** 2026-03-19

**Authors:** Rebecca J. Webb, Alexandra A. Roberts, Lee Berger, Jacques Robert, Lee F. Skerratt

**Affiliations:** ^1^ One Health Research Group University of Melbourne Melbourne Australia; ^2^ Department of Microbiology and Immunology University of Rochester Medical Center Rochester New York USA; ^3^ Canberra Health Services Garran Australian Capital Territory Australia

**Keywords:** *Batrachochytrium*, chytridiomycosis, glutathione, reactive oxygen species, ROS, virulence

## Abstract

Glutathione has important roles in diverse infections, yet its involvement in the interaction between the deadly fungal pathogen *Batrachochytrium dendrobatidis* (*Bd*) and its amphibian hosts is still unclear. Using in vitro assays and a cell infection model, we examined how glutathione influences *Bd* virulence traits and cellular host disease resistance. For *Bd*, inhibition of glutathione reductase rapidly killed zoospores, indicating that glutathione is essential for this pathogen. In addition, exposure to exogenous glutathione promoted the potential for virulence through accelerated and increased zoospore release. In host amphibian cells, *Bd* infection decreased intracellular glutathione content and increased reactive oxygen species, suggesting that chytridiomycosis pathogenesis involves oxidative stress. Depletion of host glutathione before exposure to *Bd* increased infection severity, whereas amphibian cells with slightly elevated glutathione levels were partially protected against *Bd*. However, manipulation of host glutathione levels after the establishment of *Bd* infection did not impact its intracellular growth, implying that the host glutathione‐mediated resistance only occurs during the initial *Bd* invasion process. Importantly, this effect of glutathione on host resistance is not a general response to pathogens, as it was not observed in cells exposed to the viral pathogen FV3. As glutathione increased both infectious zoospore production and host resistance to zoospore infection, our study suggests that this antioxidant may play an important role in the host/pathogen interaction during chytridiomycosis. Thus, environmental conditions and therapeutic approaches that affect glutathione systems in the host and/or pathogen have the potential to alter chytridiomycosis dynamics and should be further explored.

## Graphical abstract

Glutathione shapes interactions between *Batrachochytrium dendrobatidis* (*Bd*) and amphibian hosts. In *Bd*, glutathione reductase is essential for survival, and exogenous glutathione enhances zoospore release. In host cells, *Bd* infection depletes glutathione and increases oxidative stress. Host glutathione protects during early infection, suggesting redox balance as an important host trait.

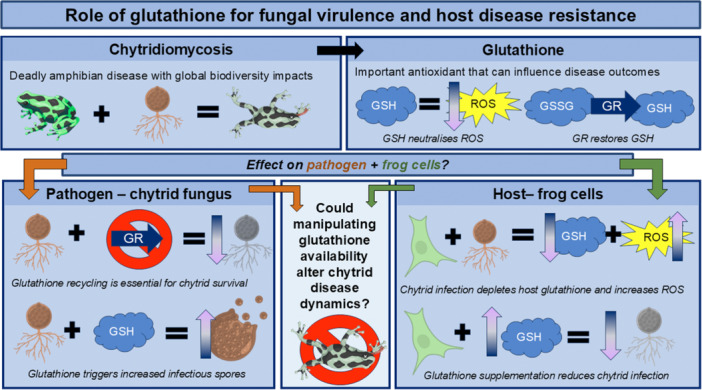

## 1 Introduction

Glutathione can influence host–pathogen interactions in multiple ways. The ubiquitous presence of glutathione in eukaryotic cells can serve as a signaling “virulence switch” for some pathogenic bacteria providing a cue that they have reached a favorable host environment (Ku and Gan 2021; Wangsanut and Pongpom 2022). This has not been described in fungal pathogens, however, and the roles of extracellular or exogenous glutathione for modulation of virulence has not been thoroughly explored (Wangsanut and Pongpom 2022). Endogenous glutathione is known to be important for fungal development, and changes in the intracellular glutathione concentration or ratio can trigger processes such as germination, conidiation, and dimorphic transition (Thomas et al. 1991; Manavathu et al. 1996; Pócsi et al. 2004). Of particular importance is the maintenance of redox state achieved via glutathione reductase (GR) mediated regulation of the ratio of reduced (GSH) and oxidized glutathione (GSSG). Without GR activity, fungi can display reduced tolerance of stress, reduced virulence, or complete inability to grow (Sato et al. 2009; Lee et al. 1997). Understanding the role of fungal glutathione systems in disease can inform new antifungal strategies (Thangamani et al. 2017).

From the host point of view, the glutathione system is required as a means to fight infection. Host‐produced glutathione is associated with resistance to viral and bacterial diseases (Herzenberg et al. 1997; Morris et al. 2013). Glutathione is also involved in defense against fungal infections, as GR‐deficient mice displayed higher *Candida* infections (Kim et al. 2019), and in cress plants, glutathione is required for resistance to the fungal pathogen *Colletotrichum gloeosporioides* (Hiruma et al. 2013).

*Batrachochytrium dendrobatidis* (*Bd*) is a fungal pathogen responsible for the disease chytridiomycosis, which has caused dramatic declines and extinctions of hundreds of amphibian species worldwide (Scheele et al. 2019). The loss of amphibians from the ecosystem has caused trophic cascade biodiversity loss (Zipkin et al. 2020) and could impact human health via reduced predation of malaria transmitting mosquitos (Springborn et al. 2022). Strategies to reduce the impact of chytridiomycosis, either by increasing host resistance or decreasing pathogen virulence, are therefore urgently needed (Berger et al. 2024). During chytridiomycosis, the fungal zoosporangia grow within amphibian epidermal cells and release infective zoospores. Mucin on the amphibian skin surface likely stimulates the waterborne zoospores to recognize the host and encyst (Robinson et al. 2022). Once inside host tissue, many putative virulence genes are upregulated in vivo (Ellison et al. 2017; Farrer et al. 2017). However, the factors that trigger these transcriptional changes during intracellular infection have not yet been resolved. We previously found that exposure to exogenous glutathione resulted in a dramatic increase in zoospore production, even in zoosporangia depleted of their endogenous glutathione supplies (Webb et al. 2023). Amphibian skin cells contain glutathione (Yang et al. 2016). However, the role that host‐derived glutathione plays in chytridiomycosis susceptibility and disease progression is unknown. If *Bd* responds to host cytosolic glutathione via accelerated and increased production of infective zoospores, this may trigger increased pathogen virulence. Conversely, amphibian glutathione may increase resistance to fungal infection, as seen in other disease systems (Ren et al. 2020). A recent study of newly metamorphosed frogs observed lower levels of glutathione in the skin and liver of *Bd*‐infected animals (Humphries et al. 2025), suggesting a role in the disease process. However, direct evidence of the impact of host glutathione availability on chytridiomycosis is lacking and requires experimental manipulation of host glutathione and quantification of disease outcomes.

The objective of the present work was to investigate the effect of host‐ and pathogen‐derived glutathione on *Bd* growth and infection. Since GR can influence the growth and virulence of other fungi, we used a potent GR inhibitor to disrupt GSSG–GSH recycling and monitored the effect on growth in culture to assess whether maintenance of the reduced:oxidized glutathione ratio is essential in *Bd*. Since exposure to exogenous glutathione can trigger virulence traits in bacterial pathogens, we exposed zoosporangia to reduced and oxidized glutathione and monitored for changes in zoospore production to understand the impact of glutathione on the *Bd* life cycle and virulence potential. To assess if this response was specifically due to glutathione, we compared these results to an alternative reducing agent (*ß*‐mercaptoethanol, BME) and an alternative extracellular amphibian skin component (mucin). A host cell infection model was used to explore how host‐derived glutathione impacts fungal virulence. We monitored the change in host glutathione content during *Bd* infection and assessed fungal infection burden when host glutathione levels were manipulated. To determine whether the effect of glutathione levels on *Bd* infection was a generalized response to pathogens, we repeated the glutathione manipulation experiments with an amphibian viral pathogen. As glutathione is often utilized in response to oxidative stress, we assessed whether reactive oxygen species (ROS) were generated during *Bd* infection and explored whether ROS originated from the host or pathogen cells.

## 2 Methods

### 2.1 Chemicals

All chemicals were purchased from Merck. GSH, GSSG, BME, mucin, cysteine, and buthionine sulfoximine (BSO) stock solutions were freshly prepared in MilliQ water and filter sterilized. The potent GR inhibitor 2‐acetylamino‐3‐[4‐(2‐acetylamino‐2‐carboxy‐ethylsulfanylthiocarbonylamino)phenylthiocarbamoylsulfanyl]propionic acid (2‐AAPA) was prepared in DMSO, and then further diluted in MilliQ water and filter sterilized to produce a 1 mM stock solution. The nonspecific ROS probe 2′,7′‐dichlorodihydrofluorescein diacetate (DCFH‐DA) was prepared as a 100 mM stock in DMSO and stored as frozen aliquots. The glutathione probe monochlorobimane (mBCI) was prepared as a 50 mM stock in DMSO and stored as frozen aliquots.

### 2.2 *Bd* Culture

Three different *Bd* isolates were used in these experiments to ensure that the impact of glutathione was not specific to a single isolate. The *Bd* isolates (#46 Waste point—*Litoria verreauxii* #5‐2013‐LB, RW, #109 Melbourne—*Lissotriton vulgaris*‐2023‐LB, and #87 Nariel Valley—*L. spenceri*‐2020‐LB) were isolated from naturally infected Australian amphibians, and all belong to the virulent Global Pandemic Lineage. Isolates were maintained in tryptone, gelatine hydrolysate, and lactose media (TGhL) following standard laboratory protocols (Prostak and Fritz‐Laylin 2021). To enable real‐time visualization of the fungal cells as they infected and grew inside frog cells, *Bd* isolate #87 was transformed with a fluorescent reporter (Webb, Vu, et al. 2024) and renamed T#5. Briefly, zoospores were electroporated to deliver a plasmid conferring tdTomato fluorescence and hygromycin resistance, and kept under hygromycin selection to produce a red fluorescing *Bd* isolate.

### 2.3 Effect of Disruption of Endogenous Glutathione on Zoospores

The potent GR inhibitor (2‐AAPA) was used to disrupt the endogenous glutathione pool by preventing the conversion of GSSG to GSH. A suspension of zoospores (#87) was collected from actively growing flasks, filtered through a 10 µM syringe filter (Millipore), centrifuged, then added to 96‐well plates at 5 × 10^4^ zoospores in TGhL per well with 10 µM 2‐AAPA, or 0.05% DMSO control. A subset of wells also contained either 1.5 mM GSH or 1.5 mM GSSG. After 30 min exposure, zoospore's survival was determined by observing movement under a microscope (a total loss of motility was considered as indicative of zoospore death). After 3 h, the exposure solution was removed and replaced with 100 µL of fresh TGhL, and incubated at 20°C. Relative growth of exposed zoospores was measured after 48 h using a methylene blue growth assay to determine the fungal biomass (Sumanasekera, Skerratt, et al. 2026) (Webb, Cuff, et al. 2024). Two‐way ANOVA and Tukey's post hoc tests were used to investigate the interaction between treatment with 2‐AAPA and supplementation with either GSH or GSSG.

### 2.4 Effect of Exogenous Glutathione on *Bd* Growth

GSH, GSSG, BME, and mucin were used to determine if they elicit a potential virulence switch in *Bd*. Standard minimal inhibitory concentrations (MIC) assays (Webb, Cuff, et al. 2024) were conducted to determine sensitivity to each chemical, and appropriate concentrations chosen for the remaining experiments. Zoospores (#46) were plated and left to encyst for 18 h as described above. Excess media was removed and replaced with 100 µL TGhL containing either GSH and GSSG (0.5–2 mM), BME (0.1–2 mM) or mucin (0.25–2.5 mg/mL) and incubated at 20°C. After an additional 48 h incubation, an aliquot (10 µL) from three replicate wells was used to quantify zoospore production using a haemocytometer. Additional time course experiments were conducted to investigate the timing and duration of zoospore production after exposure to GSH and GSSG. Zoospores were incubated for 48 h before exposure to either 2 mM GSH or 2 mM GSSG, and zoospore production calculated by counting the next generation of motile zoospores at various timepoints (24–90 h) post‐glutathione exposure. One‐way ANOVA and Dunnett's post hoc test were used to determine differences in logged zoospore count at each timepoint. The viability of zoospores produced following GSH exposure was assessed by transferring a 20 µL aliquot to a new well with 80 µL TGhL, allowing zoospores to encyst, then replacing the TGhL to remove residual traces of glutathione. Relative growth was measured via methylene blue growth assay after 48 h incubation at 20°C. One‐way ANOVA and Dunnett's post hoc test were used to determine differences in growth in zoospores obtained from GSH‐treated zoosporangia compared to those from the untreated control.

### 2.5 Effect of *Bd* Infection on Host Glutathione

To explore the role of glutathione for the host response to *Bd* infection, we took advantage of an established chytridiomycosis cell infection model utilizing commercially available immortalized amphibian “A6” cells (derived from *Xenopus laevis* kidney) (Webb, Vu, et al. 2024; Verbrugghe et al. 2019, 2025). Total glutathione was measured in A6 cells after exposure to *Bd* at two multiplicity of infection (MOI) rates. A6 cells were maintained in 75% NCTC‐109 media supplemented with 10% FCS and 2 mM glutamine at 27°C and 5% CO_2_ following standard protocols. At the beginning of the experiment, A6 cells were detached with trypsin, and added to 96‐well plates at 9 × 10^3^ cells per well. After 18 h incubation at 27°C, excess media was removed from the wells and replaced with 100 µL of solution C containing either 9 × 10^3^ (1 MOI) or 2.7 × 10^4^ (3 MOI) zoospores per well for 1 h, after which the inoculation solution as removed and replaced with 100 µL solution A (Webb, Vu, et al. 2024; Verbrugghe et al. 2019). The total glutathione concentration of host cells was measured at 4 and 24 h after infection using a commercial kit (GSH‐Glo Glutathione Assay‐Promega) following the manufacturer's directions. Two zoospore only wells were included to estimate the glutathione contribution from *Bd* cells, and this value was subtracted from the infected wells. One‐way ANOVA and Dunnett's post hoc tests were used to determine whether host cell glutathione changed in response to *Bd* infection. A separate experiment was conducted to confirm the effect of *Bd* infection using a glutathione‐specific fluorescent probe (mBCI). A6 cells were infected with 3 MOI zoospores as above and incubated at 20°C. After 72 h, excess media was removed, and cells were washed with amphibian strength PBS (APBS), and then stained with 50 µM mBCI in 70% L15 media for 1 h (Neibert and Maysinger 2012). The staining solution was removed, cells washed with APBS and each well refilled with 100 µL solution A. Glutathione was visualized under an inverted fluorescent microscope (EVOS 5000) using a DAPI filter.

### 2.6 Effect of *Bd* Infection on ROS

A similar experimental design was employed to investigate the production of ROS during infection. Initially, cells were infected with four different zoospore (#87) loads, 0 MOI, 0.5 MOI, 1 MOI, and 2 MOI, and incubated at 20°C for 4 days. ROS was visualized with using DCFH‐DA, a nonspecific redox probe that emits green fluorescence in the presence of any type of ROS. Excess media was removed and replaced with 70% L15 media containing 10 µM DCFH‐DA and incubated at 20°C for 30 min (Kim and Xue 2020), after which the solution was removed, replaced with 10 μg/mL Calcofluor White (CFW) to stain *Bd* for 5 min (Verbrugghe et al. 2019), rinsed, and replaced with 70% L15 media. Cells were imaged under GFP fluorescence to semi‐quantitatively assess ROS levels. To investigate whether ROS was host or pathogen generated, a separate experiment was conducted in which cells were infected with 1 MOI zoospores (T#5), and either: (1) supplemented with 2.5 mM cysteine on Day 3 for 8 h, (2) incubated at 27°C for 8 h, (3) supplemented with 1.5 mM cysteine and incubated at 30°C for 6 h. ROS staining was conducted as above to determine whether ROS levels decreased under these conditions. To examine the interaction between glutathione levels and ROS, infected A6 cells were first stained with 50 µM mBCI in 70% L15 media for 1 h, then stained with 10 µM DCFH‐DA for 30 min.

### 2.7 Manipulation of Host Cell Glutathione

The feasibility of manipulating host glutathione was determined using A6 cells. A6 cells were maintained in 75% NCTC supplemented with 10% FCS and 2 mM glutamine at 27°C and 5% CO_2_ as above. A6 cells were detached with trypsin, added to 96‐well plates at 6 × 10^3^ cells per well and incubated at 27°C overnight. To deplete or increase cellular glutathione, A6 cells were exposed to 10 mM BSO (Shirriff and Heikkila 2017) or 2.5 mM cysteine (Yildiz et al. 2009; Wang et al. 1997) for 18 h, respectively. Total cellular glutathione was measured using a commercial kit (GSH‐Glo Glutathione Assay‐Promega) following the manufacturer's directions. One‐way ANOVA analysis with Dunnett's post hoc tests were used to determine if total cellular glutathione responded to treatment. A separate experiment was conducted to confirm that glutathione manipulation was successful using mBCI to stain glutathione as described above. A6 cell viability upon BSO and cysteine treatment was determined using MTT assay.

### 2.8 Effect of Host Derived Glutathione on *Bd* Growth and Host Cell Health

To explore the effect of host glutathione levels on *Bd* infection, A6 cells were manipulated as above to increase or decrease glutathione levels before and after exposure to *Bd* zoospores (T#5). Actively growing A6 cells were detached with trypsin, added to 96‐well plates at 2.5 × 10^4^ cells per well and incubated with 10 mM BSO or 2.5 mM cysteine to alter glutathione levels. After 18 h incubation at 27°C, excess media was removed from the A6 wells and replaced with 100 µL of solution C containing 5 × 10^4^ zoospores (2 MOI). After 2 h incubation at 20°C, the zoospore solution was removed and replaced with 100 µL of solution A, along with either 10 mM BSO or 0.25 mM cysteine and incubated at 20°C. Cysteine concentrations were reduced after *Bd* introduction as MIC experiments in TGhL indicated that concentrations above 1 mM affect *Bd* growth, whereas BSO does not inhibit *Bd* growth until 30 mM (Webb et al. 2023). After 24 h, the media was replaced with solution A, and the cells left to grow for an additional 6 d. To understand the effect of host cell glutathione on existing infections, additional experiments were conducted in which A6 cells were supplemented with 2.5 mM cysteine at 24 h postinfection. *Bd* growth was assessed by taking three randomly chosen images at 40× magnification fields of view (FOV) per well under red fluorescence, and calculating the total area occupied by the red fluorescing *Bd* cells using ImageJ relative to the control average. If wild‐type *Bd* was used to infect the cells, *Bd* growth was measured using CFW (Verbrugghe et al. 2019). *Bd* zoospore production was measured by counting the number of motile zoospores present at three randomly chosen FOV per well and calculated relative to the control average. A6 cell health was determined by staining with 50 µL sterile filtered 0.5 µg/mL DAPI (Invitrogen) for 5 min, after which the staining solution was removed and replaced with 100 µL solution A (Sumanasekera, Berger, et al. 2026). Uninfected controls were included to check for damage from cysteine or BSO treatment alone. Host cell damage was measured by imaging under blue fluorescence and the number of DAPI‐positive cells was calculated using ImageJ, relative to the untreated control average, using the uninfected treatment as a blank. One‐way ANOVA analysis with Dunnett's post hoc tests were used to determine if *Bd* growth, zoospore production, or host cell damage differed from untreated control cells.

### 2.9 Effect of Host Derived Glutathione on Disease Susceptibility of Host Cells to Frog Virus 3

To explore whether changes in host cell glutathione cause a generalized effect on disease susceptibility, the above experiment was repeated with a ranavirus as an alternative amphibian pathogen. After 18 h BSO and cysteine incubation, excess media was removed, replaced with 100 µL A6 media containing 0.6 MOI Frog Virus 3 (FV3) (De Jesús Andino et al. 2024), and incubated at 30°C for 1 h. After viral exposure, excess media was removed and replaced with fresh A6 media containing 10 mM BSO or 2 mM cysteine and incubated at 27°C for 72 h. At 56 h postinfection A6 cell survival was measured using a CyQUANT MTT Cell Proliferation Assay Kit (Thermo) following the manufacturer's instructions and calculated relative to the FV3 only control.

### 2.10 Statistical Analysis

All data were obtained from in vitro experiments in 48‐ or 96‐well plates, with each individual well considered a pseudo‐biological replicate. Multiple measurements (e.g., counts or images) from the same well were treated as technical replicates and averaged. Data were analyzed using GraphPad Prism 10.2.3. Count data (zoospore counts and damaged cell counts) were logged transformed. One‐way, two‐way, or repeated ANOVA and appropriate post hoc tests were used to identify significant differences, with a significance level set at *p* < 0.05. All test assumptions of normality were met. Graphical data are expressed as mean ± standard deviation.

## 3 Results

### 3.1 Decreased GSH Levels via Inhibition of Glutathione Reductase Is Acutely Toxic to *Bd*

**Figure 1:**
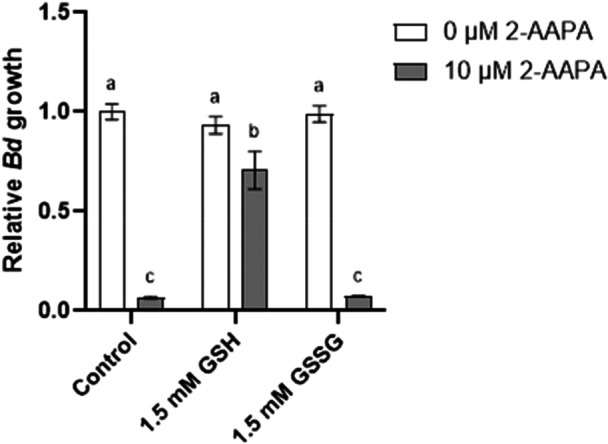
The toxic effects of 2‐AAPA can be rescued by supplementation with GSH, but not GSSG. 2‐AAPA is a potent glutathione reductase inhibitor, preventing the conversion of endogenous oxidized glutathione (GSSG) back to the reduced form (GSH). *Bd* zoospores were incubated with 10 µM AAPA with or without GSH or GSSH. Bars show mean growth and SD of three replicate wells measured 24 h after 2‐AAPA exposure using a methylene blue growth assay. Bars that do not share a common superscript letter differ significantly according to a two‐way analysis of variance (ANOVA) for independent samples, followed by Tukey's post hoc test (*p* < 0.05), *n* = 18.

We have previously found that chronic inhibition of endogenous glutathione synthesis prevents *Bd* growth and development in vitro (Webb et al. 2023). Here we found that inhibition of the enzyme responsible for endogenous glutathione recycling (GR) is acutely toxic (Figure 1). There was a significant interaction between 2‐AAPA treatment and the type of glutathione supplementation (ANOVA *F*_2,12_ = 104.7, *p* ≤ 0.0001). Exposure to 10 µM 2‐AAPA caused rapid toxicity to zoospores, resulting in a rapid loss of motility and significantly less growth than the untreated control (*p* ≤ 0.0001). In 2‐AAPA treated wells, supplementation with 1.5 mM GSH provided a partial rescue effect, with growth significantly higher than the nonsupplemented 2‐AAPA treated control (*p* ≤ 0.0001), whereas 1.5 mM GSSG did not reverse the effects of 2‐AAPA exposure and growth was not significantly different to the nonsupplemented 2‐AAPA treated control (*p* ≥ 0.9999).

### 3.2 Exogenous Glutathione Triggers Increased Zoospore Production in *Bd*

**Figure 2:**
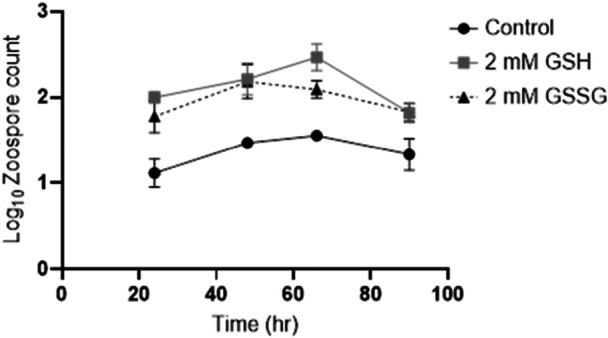
Increased zoospore production is sustained after exogenous glutathione exposure. Zoospore production of mature (2 d old) *Bd* zoosporangia exposed to 2 mM reduced (GSH) or oxidized (GSSG) glutathione compared to an untreated control over one life cycle. Mean logged zoospore counts estimated via haemocytometer from two replicate wells per treatment per timepoint and SD, *n* = 24.

**Figure A1:**
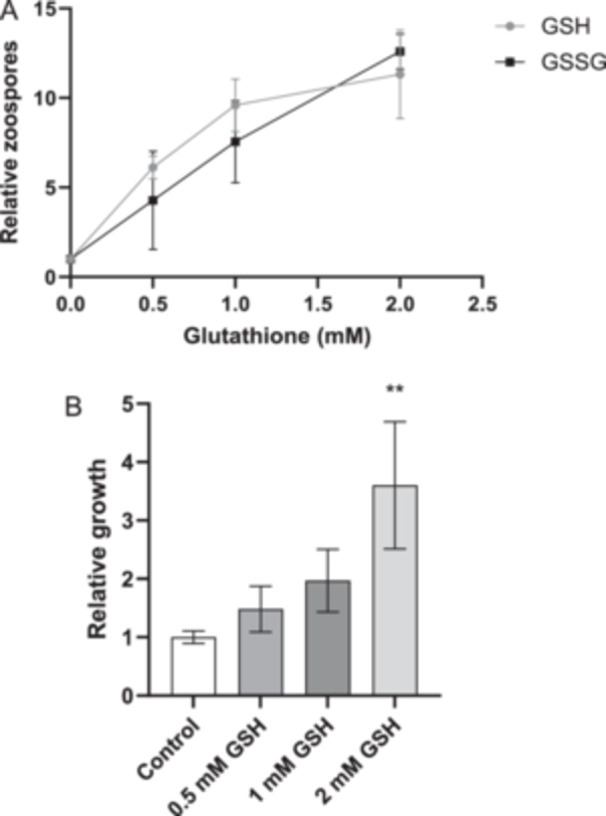
Exogenous glutathione exposure increases zoospore production. (A) Young (20 h) zoosporangia (#46) exposed to either reduced (GSH) or oxidized (GSSG) glutathione produced more zoospores compared to control cells. Zoospores from three replicate wells per treatment were counted on a hemocytometer 48 h after glutathione exposure and calculated relative to the control. Mean zoospores per glutathione concentration and SD are displayed, *N* = 12. (B) To check for the viability of these zoospores, an aliquot (20 µL) of these zoospores was transferred to a new plate. Zoospores released from glutathione‐exposed zoosporangia encysted and grew. Bars show growth and SD of three replicate wells measured at 48 h since plating using a methylene blue growth assay, relative to the control. One‐way ANOVA and Dunnett's post hoc test were used to determine differences from the untreated control, (**p ≤ 0.01), *n* = 12.

To investigate potential virulence triggers, young zoosporangia (20 h) were exposed to GSG, GSSG, BME, or mucin and monitored for zoospore release at various timepoints after exposure. Both oxidized and reduced glutathione triggered an increase in viable zoospore production relative to the untreated control (Figure A1). An increase in zoospore production was not observed in zoosporangia exposed to BME (another thiol reducing agent), or mucin (a component of the amphibian skin mucosome). To substantiate this finding, mature zoosporangia (48 h) were exposed to different concentrations of GSH or GSSG and zoospore release monitored over time (Figure 2). Glutathione appeared to act as a catalyst that accelerated zoospore release by at least 12 h compared to the control, and this increased zoospore production was sustained during the growth cycle (Figure 2). There were significantly more zoospores released by zoosporangia exposed to GSH or GSSG at 24 h (*p* = 0.0155, 0.0342, respectively), 48 h (*p* = 0.0310, 0.0344, respectively), 66 h (*p* = 0.0063, 0.0279, respectively). By 90 h post‐exposure, zoospore release was not significantly different to that of the control.

### 3.3 *Bd* Infection Depletes Host Glutathione Stores and Increases ROS

**Figure 3:**
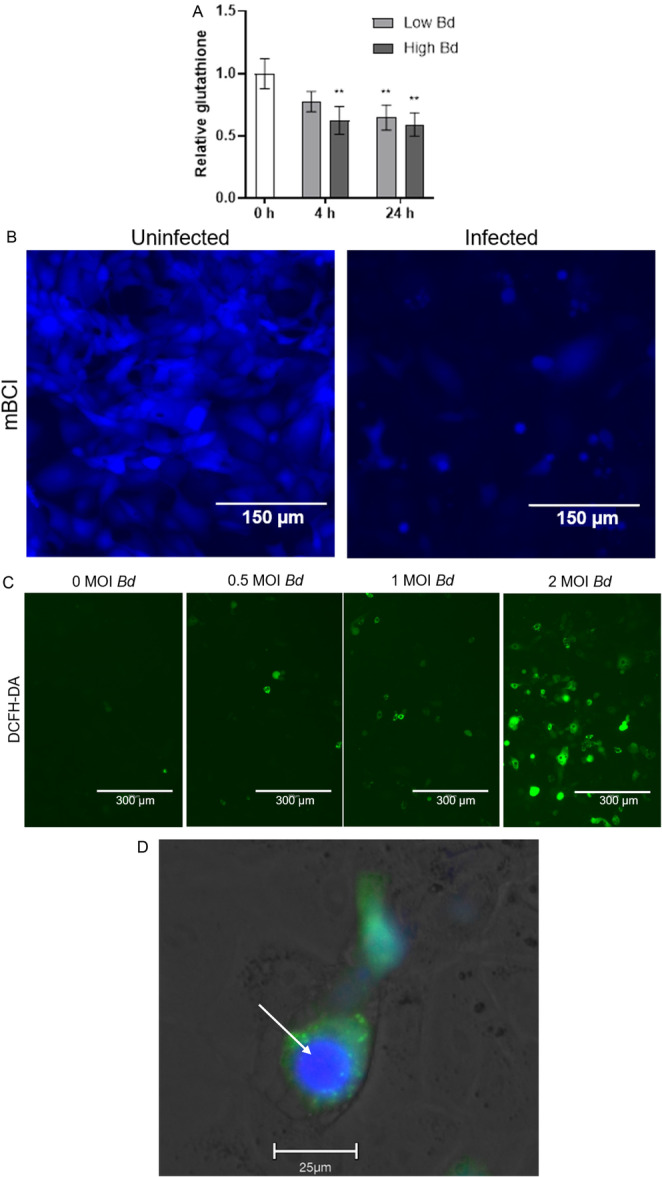
*Bd* infection causes a decrease in host cell glutathione content and an increase in ROS. (A) The total glutathione abundance of immortalized amphibian cells (A6) was measured with a luminescent GSH assay (Promega) before and during infection with either a low (1 MOI, multiplicity of infection) or high (3 MOI) concentration of zoospores. Mean total glutathione levels and SD of 3 replicate wells per treatment and timepoint are displayed. One‐way ANOVA was used to determine significant differences from the 0 h control timepoint indicated by asterisks (**p ≤ 0.01), *n* = 15. (B) Glutathione content of uninfected and infected A6 cells was visualized using 50 µM monochlorobimane (mBCI). Representative image at 200× magnification. (C) The ROS content of uninfected A6 cells compared to cells infected with 0.5 MOI, 1 MOI, or 2 MOI zoospores was visualized using 2′,7′‐dichlorodihydrofluorescein diacetate (DCFH‐DA), representative images at 100× magnification. (D) Combining both stains indicates that *Bd* zoosporangia (arrow) stain strongly for glutathione (blue) compared to the host cells which stain strongly for ROS (green).

**Figure A2:**
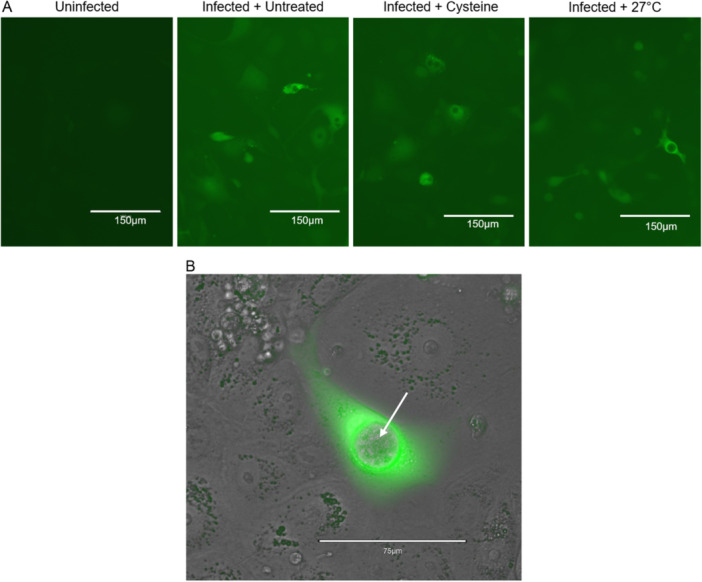
Intracellular ROS appears to be host generated. (A) The amount of ROS in infected A6 cells did not decrease in response to supplementation with 2.5 mM cysteine or incubation at 27°C. Uninfected cells and untreated infected cells for comparison. ROS was visualized using 2′,7′‐dichlorodihydrofluorescein diacetate (DCFH‐DA), representative images at 100× magnification. (B) Close‐up image to demonstrate that ROS persists after inactivation of *Bd*. Infected A6 cells (D5 post infection) were supplemented with 1.5 mM cysteine and incubated at 30°C for 6 h, before staining DCFH‐DA for 30 min to visualize ROS. Arrow shows intercellular *Bd* zoosporangia, representative image at 400×, scale bar is 75 µm.

An in vitro cell infection model (Webb, Vu, et al. 2024; Verbrugghe et al. 2019) was used to further investigate the role of glutathione during chytridiomycosis. Infected amphibian (A6) cells had decreased total glutathione levels compared with uninfected controls (Figure 3A). Using a luminescent glutathione assay, we found exposure to low zoospore burdens significantly decreased host glutathione content by 36% after 24 h (*p* = 0.0058). At high zoospore burdens, host glutathione content was significantly decreased by 38% after 4 h (*p* = 0.0038) and 41% after 24 h (*p* = 0.0021). Visualization of glutathione content using 50 µM mBCI produced similar results, with infected cells displaying lower staining intensity (Figure 3B). Infection also increased amphibian cell ROS in a dose‐dependent manner (Figure 3C). ROS appeared to be closely associated with *Bd* infection, as DCFH‐DA staining was concentrated in cells containing large intracellular zoosporangia (Figures 3D and A2). Staining shows *Bd* zoosporangia contained more glutathione and less ROS compared to the A6 cells (Figure 3D).

To investigate whether the ROS was generated by the host or the pathogen, we inhibited *Bd* growth by increasing the temperature beyond the thermal optimum of *Bd*, and/or supplemented the cells with cysteine (a glutathione precursor) with the hypothesis that if ROS was pathogen generated it should decrease under these conditions. None of these treatments resulted in decreased intracellular ROS (Figure A2). Together, these results suggest that the observed ROS is likely generated by the host cells rather than *Bd*.

### 3.4 Host Glutathione Availability Affects Susceptibility to *Bd* but Not Frog Virus 3 (FV3)

**Figure 4:**
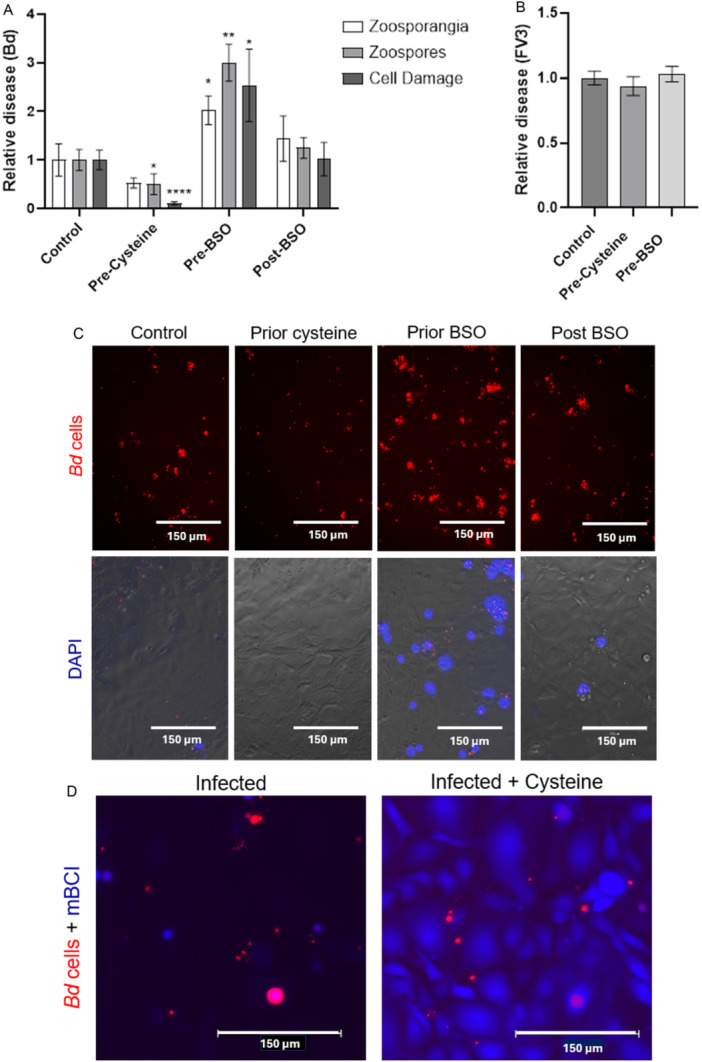
Glutathione availability in host cells affects chytridiomycosis. Immortalized amphibian cells (A6) with varying glutathione concentrations were exposed to *Bd* zoospores or Frog Virus 3 (FV3) to assess differences in host cell damage and *Bd* infection burden as measures of disease. Glutathione content of A6 cells was increased prior to *Bd* infection via supplementation with 2.5 mM cysteine, and depleted either prior or after infection via addition of 10 mM BSO. (A) *Bd* growth was measured as the red fluorescence surface area (zoosporangia) or zoospore count (zoospores) at 7 d. A6 cell damage was assessed by the number of DAPI‐positive cells at 3 d. Mean values from three replicate wells and SD are displayed, One‐way ANOVA and Dunnett's post hoc tests were used to determine significant differences from the control (*p = ≤ 0.05, **p ≤ 0.01, ****p ≤ 0.0001), *n* = 12. (B) A similar experiment in which A6 cells were exposed to 2.5 mM cysteine or 10 mM BSO before challenge with FV3 ranavirus. Relative disease was calculated by MTT assay at 3 d. Mean cell activity from three replicate wells and SD are displayed, *n* = 12. (C) Representative images of *Bd* infection show *Bd* growth (red) after 7 d, or DAPI‐positive A6 cells (blue) after 72 h. Cells received no treatment (control), incubation with 2.5 mM cysteine prior to infection (prior cysteine), incubation with 10 mM BSO prior and after infection (prior BSO), or incubation with 10 mM BSO only after infection (post‐BSO). (D) Supplementation with cysteine 24 h after *Bd* infection restored depleted glutathione levels, as indicated with mBCI staining, but did not reduce *Bd* growth. Representative images at 200× show glutathione (blue) and *Bd* cells (red) 4 days after infection.

**Figure A3:**
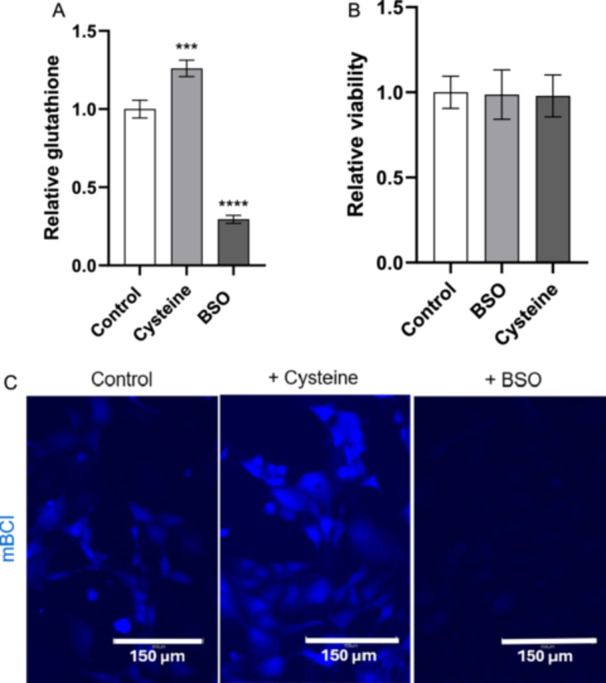
Manipulation of glutathione concentrations in A6 cells. A6 cells were incubated with 2.5 mM Cysteine (a glutathione precursor), or 10 mM buthionine sulfoximine (BSO) (a glutathione synthesis inhibitor) for 18 h. (A) Bar graphs show mean and SD of total glutathione levels were measured using a luminescent quantification assay (Promega), calculated relative to the control. One‐way ANOVA and Dunnett's multiple comparison test were used to determine significant differences in glutathione content from the control, (***p ≤ 0.001, **** *p* ≤ 0.0001), *n* = 9. (B) One‐way ANOVA indicated this treatment did not affect A6 cell viability as determined by MTT assay, *n* = 12. (C) For further confirmation, mBCI was used to visualize glutathione content in A6 cells. Representative images at 200×.

**Figure A4:**
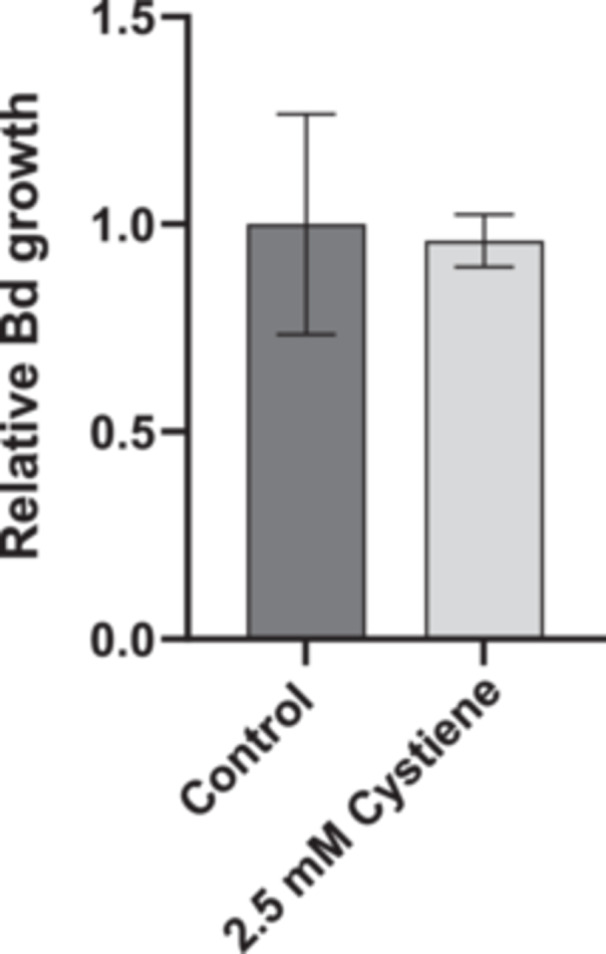
Glutathione supplementation of infected cells does not reduce growth. A6 cells were infected with *Bd* zoospores and infected allowed to progress for 24 h, after which cells were supplemented with 2.5 mM cysteine for 3 days. *Bd* growth was quantified by staining with Calcofluor White and the relative area of stained *Bd* measured using ImageJ from the average of two images per well of three replicate wells, *n* = 6.

We achieved manipulation of glutathione levels in A6 cells using BSO and cysteine. Total glutathione concentrations decreased by ~70% after incubation with 10 mM BSO and increased by ~25% after incubation with 2.5 mM cysteine for 18 h, and these effects were not due to impacts on A6 viability (Figure A3). To investigate the effect of host cell glutathione concentration on disease parameters, we infected cells that had been preincubated with either BSO or cysteine and compared *Bd* growth to that within untreated cells. To distinguish whether host cell glutathione concentration is important during initial infection or subsequent *Bd* growth, an additional treatment group included A6 cells that were only incubated with BSO immediately after infection. The availability of glutathione in host cells impacted *Bd* loads (zoosporangia and zoospores) as well as host cell damages (Figure 4A,C). Host cells with elevated glutathione had significantly less zoospores present compared to control cells (*p* = 0.0194). In contrast, host cells with depleted glutathione stores prior to infection supported significantly more *Bd* growth (*p* = 0.0.125) and zoospore production (*p* = 0.0018) compared to the control. Host cells that experienced glutathione depletion only after *Bd* zoospore encystation did not differ from the untreated control (Figure 4A,C). To investigate the effect of glutathione on host cell health during infection, we assessed host cell damage using DAPI (Sumanasekera, Berger, et al. 2026), which stains the nuclei of cells with compromised membranes (Boroda et al. 2016). The relative number of DAPI‐stained cells followed a similar pattern to *Bd* growth (Figure 4A,C). Manipulation of host cell glutathione with BSO or cysteine did not change A6 cell survival when exposed to an alternative amphibian pathogen, FV3 (Figure 4B). To investigate the effect of glutathione on existing intracellular infections, we supplemented A6 cells with 2.5 mM cysteine 24 h after infection. Cysteine supplementation restored glutathione levels in infected cells (Figure 4D) but had no significant effect *Bd* growth (Figure A4).

## 4 Discussion

Our study provides compelling evidence that glutathione plays a fundamental role in the pathogenesis of chytridiomycosis, impacting traits of both the pathogen and the host. We also show for the first time that ROS increases within infected cells in vitro.

Exposing *Bd* to glutathione in vitro triggered a dramatic and sustained increase in zoospore production, indicating that glutathione, ubiquitous within host cells, may act as a cue to signal a favorable host environment. We also show that the maintenance of redox balance via glutathione recycling is an essential process in *Bd*, as inhibition of GR activity is acutely toxic. For the host, increased glutathione availability promotes resistance to *Bd* infection, possibly due to its role in protecting the host from ROS generated in response to fungal invasion.

### 4.1 Effect of Glutathione on *Bd*

In vitro, *Bd* zoosporangia responded to low levels of exogenous glutathione via a dramatic and sustained increase in zoospore production. Preliminary experiments indicated that the concentration of GSH needed to trigger increased zoospore release varied slightly depending on the *Bd* isolate. The timing of glutathione exposure does not appear to be crucial, as zoosporangia responded in a similar manner when exposed at 24 or 48 h. Accelerated zoospore production has been correlated with high virulence strains in in vivo infection trials (Fisher et al. 2009; Voyles 2011; Langhammer et al. 2013; Davidson et al. 2025; Greener et al. 2020), thus, our results suggest that glutathione is an important virulence factor for infection by *Bd*. The mechanism behind the stimulation of zoospore release by glutathione is not yet clear, but the observation that both GSH and GSSG elicit a zoospore response, whereas mucin or BME does not, implies that *Bd* is not merely responding to exposure to host tissue or a generalized redox change, as hypothesized in *Burkholderia pseudomallei* (Wong et al. 2015). Previous studies have demonstrated that zoosporangia under severe endogenous glutathione depletion also increase zoospore production above baseline when exposed to exogenous glutathione (Webb et al. 2023). Thus, exogenous glutathione may be acting as a specific trigger to spur accelerated zoospore release. Perhaps, glutathione serves as a transcriptional activator of growth‐associated genes, or increased glutathione may facilitate differential glutathionylation of proteins essential for metabolism such as the glycolytic enzyme enolase (Fratelli et al. 2002). In human T lymphocytes, the glycolytic enzyme enolase is constitutively glutathionylated, which reduces its enzymatic activity. Peroxide stress increases glutathionylation of human enolase to reduce activity and likely protects the lymphocytes against oxidative stress by reducing metabolism (Fratelli et al. 2002). In *Bd*, enolase has also been shown to be constitutively glutathionylated. However, in contrast to human lymphocytes, peroxide stress reduces the proportion of glutathionylated enolase in *Bd* (Claytor 2020). Further studies are required to confirm whether changes in redox balance, and de‐glutathionylation of enolase specifically, stimulate the glycolysis pathway and *Bd* growth.

Given the likely association of glutathione with *Bd* virulence, we sought to investigate the role of endogenous glutathione in *Bd*. Continual synthesis of endogenous glutathione is essential for normal growth and development (Webb et al. 2023), but the importance of maintaining the correct ratio of reduced:oxidized glutathione in *Bd* was unknown. To examine the importance of this process, we took advantage of the potent *GR* inhibitor 2‐AAPA (Seefeldt et al. 2009), commonly used at 25 µM (Gansemer et al. 2020). Inhibition of GR prevents *Bd* from converting GSSG into GSH, and from our previous studies on GSH depletion, we predicted that 2‐AAPA would have some impact on growth and metal tolerance (Webb, Cuff, et al. 2024). Unexpectedly, preventing glutathione recycling was acutely toxic to *Bd*. As opposed to inhibiting glutathione synthesis via BSO, which did not affect zoospores, and took at least 24 h to impact growth of zoosporangia (Webb et al. 2023), inhibiting GR with 10 µM 2‐AAPA killed zoospores within 30 min, and 50 µM 2‐AAPA killed zoosporangia within a few hours. To confirm that the toxicity was due to depletion of GSH, we supplemented 2‐AAPA treated zoospores with either GSH or GSSG (Sato et al. 2009). GSH, but not GSSG, reversed the effect of 2‐AAPA, confirming the mechanism of toxicity as redox imbalance and the inability to recycle GSH from GSSG. To our knowledge, this is the first time that the use of 2‐AAPA has been validated in chytrid fungi. This demonstrates that GSH and the maintenance of the redox balance are essential in *Bd*. The acute requirement for glutathione reductase activity in *Bd* is in contrast with other fungal species, in which GR knockouts display only reduced tolerance to oxidative stress (Muller 1996) or temperature (Sato et al. 2009). Given that *Bd* cannot survive even a few hours without the ability to recycle glutathione, GSH represents an important molecule for *Bd*. As such, GR is an indispensable enzyme and potential target for novel antifungal strategies such as RNAi (Berger et al. 2024).

### 4.2 Effect of Glutathione on Host Cells

Glutathione also functions in protecting amphibian cells against *Bd* infection. In an A6 cell infection model, we saw increased fungal growth when host cells were deprived of glutathione via BSO at the time of infection. When A6 glutathione levels were increased via cysteine supplementation, there was a reduction in *Bd* and associated host cell damage. Since *Bd* growth in vitro is increased with additional glutathione, the opposite intracellular effect suggests the outcome of manipulating cell glutathione levels is a result of altered host resistance. This is consistent with host resistance to other fungal pathogens. We explored the precise timing of glutathione's role in immunity by depleting glutathione in A6 cells before and after infection with *Bd*. Indicators of disease increased in A6 cells depleted prior to—but not after—zoospore exposure, indicating that host cell glutathione is important during initial zoospore infection. We note that the effect of host cell glutathione was not observed when A6 cells were exposed to a viral pathogen, suggesting that this is not a generalized disease response, but may be specific to chytridiomycosis. As glutathione levels were lower in *Bd*‐infected A6 cells, this suggests host glutathione is being utilized for immune defense. Alternatively, *Bd* may be modulating the host glutathione to its own advantage as occurs during *Cryptococcus* infections (Black et al. 2024).

To further investigate glutathione relevance for host resistance to *Bd* infection, we monitored cellular ROS, and found it increased in infected A6 cells. The response correlated with zoospore dose. However, it was not clear whether ROS was produced by *Bd* as a virulence mechanism, as seen in many phytopathogenic fungi (Wang et al. 2020; Zhang et al. 2020), or alternatively produced by the host as a response to infection (James et al. 2024), or associated membrane damage (Ren et al. 2020). To help resolve this, we attempted to decrease *Bd* metabolic activity by increasing the temperature outside of its thermal optimum. In addition, we supplemented the host cells with cysteine to increase the capacity to neutralize unwanted ROS. Neither of these treatments resulted in a decrease in ROS. This preliminary evidence suggests that the observed high levels of ROS are generated by the host rather than the pathogen. As DCFH‐DA is a general ROS probe, the use of more specific probes or NOX‐inhibitors in the future would help to further understand the response of *Bd* infection on host redox status.

Combined, these results suggest that glutathione is an important host resistance mechanism, but that it may also be important for *Bd* to withstand ROS. Indeed, the ability to scavenge host produced ROS is a virulence factor in many phytopathogenic fungi, and disruption of oxidative stress enzymes, such as superoxide dismutase (SOD), can result in lower virulence (Ding et al. 2024). This may also be the case for *Bd*, as SOD and glutathione transferase (GST), are differentially expressed between *Bd* pandemic and nonpandemic lineages (McDonald et al. 2020), suggesting that managing ROS could be related to virulence. Glutathione is utilized by *Bd* in response to oxidative stress (Webb et al. 2023), and we observed that *Bd* cells stain strongly with mBCI, indicating they contain high concentrations of glutathione. We also found that GR (needed to recycle GSSG back to GSH during ROS detoxification) is essential for zoospore viability. Together, these results point to an important role for ROS detoxification in *Bd*. Future functional genetic studies could provide direct evidence of the importance of these enzymes during the infection process and provide targets for new antifungal strategies.

Studies in other fungal pathogens have shown the potential of glutathione modulation for biocontrol. Direct supplementation with glutathione reduced cell mortality in a *Candida albicans* in vitro infection model (Ren et al. 2020). In crops, application of chemicals (Bolter et al. 1993) or genetic constructs (Ding et al. 2024) that increase GSH levels can provide strong plant protection against fungal pathogens. We found that frog cells supplemented with cysteine were able to restore glutathione levels and reverse the depletion caused by *Bd* infection. Future studies should investigate whether glutathione supplementation can reduce chytridiomycosis in vivo. Given that amphibian cellular glutathione can be reduced by environmental contaminants such as agricultural pesticides (McMahon et al. 2011; Jiménez et al. 2021; Van Meter et al. 2022), their potential immunosuppressive effects regarding chytridiomycosis should be investigated. In contrast, exposure to environmental heavy metals can increase glutathione in amphibian tissues (Prokić et al. 2016), perhaps contributing to increased survival in “metal disease refuges” (Esmaeilbeigi et al. 2024). Data are needed on the glutathione concentration of amphibian epidermis, to compare to levels that impacted growth in the kidney cell cultures. Further work could also test for correlations in susceptibility to chytridiomycosis and variation in epidermal glutathione concentrations between individuals or amphibian species.

## 5 Conclusion

The current body of knowledge suggests a complex relationship between *Bd* and the glutathione system. Glutathione is important for *Bd* growth (Webb et al. 2023) and metal tolerance (Webb, Cuff, et al. 2024). Given the role of GR in glutathione recycling is essential for zoospore survival, targeting this enzyme could provide an opportunity for developing a novel antifungal strategy. Glutathione also plays an important role in host disease resistance, as infection causes depletion of intracellular glutathione, while host cells with elevated glutathione levels are less susceptible to *Bd* infection. Therefore, increasing host glutathione levels may represent an opportunity to improve resistance to chytridiomycosis. We discovered that ROS increases in host cells during infection, and these ROS are likely generated by the host rather than from the pathogen itself. Glutathione's role as an antioxidant likely modulates this process, thus contributing to host resistance. The fact that *Bd* zoosporangia (the parasitic intracellular life stage) contain high levels of glutathione would help the pathogen to resist ROS activity from the host. Taken together, these results provide evidence that glutathione plays a complex role in the interplay between pathogen, host, and environment in the pathophysiology of chytridiomycosis. Future work is needed to evaluate whether modulation of host and/or pathogen glutathione systems represents a viable strategy for disease intervention.

## Author Contributions

**Rebecca J. Webb:** conceptualization (lead), methodology (lead), investigation (lead), formal analysis (lead), funding acquisition (equal), writing – original draft (lead). **Alexandra A. Roberts:** conceptualization (supporting), methodology (supporting), supervision (equal), writing – reviewing and editing (equal). **Lee Berger:** methodology (supporting), supervision (equal), writing – reviewing and editing (equal), funding acquisition (equal), resources (equal). **Jacques Robert:** methodology (supporting), supervision (equal), writing – reviewing and editing (equal), resources (equal). **Lee Skerratt:** formal analysis (supporting), supervision (equal), writing – reviewing and editing (equal), funding acquisition (equal), resources (equal).

## Ethics Statement

The authors have nothing to report.

## Conflicts of Interest

The authors declare no conflicts of interest.

## Data Availability

The data that support the findings of this study are openly available in Melbourne Figshare at https://figshare.unimelb.edu.au/.
